# Extremely Distant and Incredibly Close: Physical Proximity, Emotional Attachment and Caregiver Burden

**DOI:** 10.3390/ijerph19148722

**Published:** 2022-07-18

**Authors:** Eva Bei, Karin Mashevich, Orit Rotem-Mindali, Shira Galin-Soibelman, Ofra Kalter-Leibovici, Tami Schifter, Noa Vilchinsky

**Affiliations:** 1 Department of Psychology, Faculty of Social Sciences, Bar-Ilan University, Ramat Gan 5290002, Israel; mashevk@biu.ac.il (K.M.); shiragalin@gmail.com (S.G.-S.); noa.vilchinsky@biu.ac.il (N.V.); 2 Department of Geography and Environment, Bar-Ilan University, Ramat Gan 5290002, Israel; orit.rotem@biu.ac.il; 3 The Gertner Institute, Sheba Medical Center, Ramat Gan 5290002, Israel; ofrakl@gertner.health.gov.il (O.K.-L.); tami@camoni.co.il (T.S.); 4 Sackler Faculty of Medicine, Tel Aviv University, Tel Aviv P.O. Box 39040, Israel

**Keywords:** informal care, physical proximity, attachment orientations, caregiver burden

## Abstract

Informal caregivers are at risk of caregiver burden, and physical proximity to the care recipient may add to this negative outcome. Yet, individual differences in emotional proximity to the care recipient such as attachment orientations may contribute to caregivers’ comfort towards different degrees of physical proximity, leading to varying levels of burden. The current study is the first to explore the role of physical proximity on caregiver burden as moderated by attachment orientations. A sample of 162 Israeli caregivers who are active users of the Camoni website completed our online survey. Sociodemographic characteristics, including a self-reported questionnaire on the physical proximity to the care recipient, were collected. Caregivers’ attachment orientations were assessed with the Experiences in Close Relationships–Relationship Structures questionnaire. Caregiver burden was assessed using the Caregiver Burden Inventory. Multiple regression and simple slope analyses were conducted. Attachment anxiety and avoidance were positively associated with burden, whereas physical proximity was not. Attachment avoidance, but not attachment anxiety, moderated the association between physical proximity and caregiver burden, with caregivers who live closer to their care recipient experiencing greater burden when high levels of avoidance were present. Our findings reveal the complex dynamics between attachment orientations and physical proximity in the context of informal care, highlighting the need for better integration of these two interlinked constructs in both care research and practice.

## 1. Introduction

With the growth in ageing populations and increasing prevalence of chronic illness, the number of care-dependent adults is rising [1,2,3,4,5]. Informal caregivers, who provide unpaid care to family members or friends with a chronic illness, play a vital societal role in sustaining long-term care settings and maintaining outpatient care [6,7]. Caregiving encompasses a wide variety of activities and tasks including social and emotional support, assistance with activities of daily living (e.g., bathing, toileting) and household tasks, management of financial affairs and monitoring of healthcare services [7].

Caregivers can experience both positive and negative outcomes as a result of their caretaking responsibilities [8,9,10]. Informal care can lead to an enhanced relationship with the care recipient and feelings of personal growth and satisfaction for the care provided and the skills obtained [8,11,12,13]. At the same time, many caregivers are at risk of experiencing adverse health and psychosocial effects related to their caregiving role, such as poor physical and mental health [13,14,15,16,17], financial problems [18], loneliness and social isolation [19]. A negative appraisal of the caregiving situation can increase caregiver burden, an experience described as a multidimensional response to the physical, psychological and emotional toll of caring [15,16,17].

### 1.1. Physical Proximity and Caregiving

The physical proximity between caregivers and their care recipient is an important structural factor that might determine the care provided and shape the caregiving experience [20,21]. In recent decades, demographic and social changes such as increased societal mobility and population migration have affected the traditional patterns of providing informal care [20,21]. While several caregivers continue to live with their care recipient, many others provide care from a geographic distance [21,22]. 

Geographically diverse caregiving samples may present with different caretaking responsibilities and unique needs and burdens associated with the physical proximity to their care recipient. Prior studies have found that those who live with their care recipient or very close to them experience high levels of burden and emotional distress, potentially resulting from greater competition between caretaking responsibilities and other time-demanding activities when co-residing or living within a proximal distance [23,24]. On the other hand, geographic distance can create additional burdens for what is already often stressful care work. In the few studies conducted, distance caregivers (DCGs) reported high levels of burden and emotional distress related to feelings of inadequacy on how to assess the needs of their loved one from afar and uncertainty regarding the progression of their illness [21,25]. Experiencing the added challenges of caring from afar—such as the need of travelling back and forth to provide hands-on care to their loved one—DGCs also reported that caring was taxing on their finances, job accomplishments and social and familial relationships [26,27]. 

Despite evidence on the unique complications and challenges experienced by geographically diverse caregiving populations, research on the role of physical proximity on caregiver burden remains scarce. In their recent study, Li et al. found that DCGs who live more than 30 min away from their frail elderly parents are more distressed by subjective burden than those who co-reside or live closer to the care recipient [21]. However, another comparative study found that caregivers who live closer to the care recipient experience greater levels of social and emotional strain [28]. 

### 1.2. Emotional Proximity and Caregiving

Individual differences among caregivers, and particularly differences in personal characteristics related to the ability and willingness to perform the caregiving role, may affect caregivers’ comfort and appraisals towards their physical proximity or distance from the care recipient, leading to varying degrees of caregiver burden [6]. One such personal characteristic is attachment orientation. According to the theory of attachment, during the early years of life, an emotional bond is formed between infants and their primary caregivers [29]. This early attachment towards the primary caregiver influences interpersonal relationships and individuals’ ability to form and maintain new emotional bonds with others later in life [29,30,31]. Attachment is commonly conceptualized as two orientations: attachment anxiety (“hyper-activation of the attachment system”) and attachment avoidance (“deactivation of this system”). Anxious–ambivalent people are clingier in their relationships and worry that the attachment figure will not be available in times of need, whereas avoidant people prefer not to rely on others, seeking independence and avoiding intimate and close relationships [31]. Individuals who score low on both orientations are considered securely attached. Securely attached individuals tend to be more comfortable with their significant others and feel competent in their ability to regulate affect in stressful situations [31].

A major stressor such as a chronic health condition of a significant other is likely to activate the attachment system, shaping caregivers’ reaction to the care recipient’s needs [32,33]. Individuals with greater attachment security tend to provide informal care that is sensitive, cooperative, and warm [6]. Conversely, insecurely attached individuals are less likely to provide sensitive care. For example, avoidant individuals’ rigid self-reliance and discomfort with closeness seem to moderate their ability to provide responsive caregiving. Anxious caregivers tend to provide a kind of care that is focused more on self-needs than their care recipient’s needs, perhaps due to their own anxiety of receiving less attention than their significant other who is ill [6]. Empirical findings have also indicated that insecure attachment patterns of caregivers positively correlate with poorer mental health [34,35,36,37,38,39]. Specifically, higher attachment anxiety has been related to higher levels of caregiver burden [37,38], depression and anxiety [36,37], whereas attachment avoidance has been positively related to health problems [38] and negatively related to self-esteem [36,39]. In contrast, higher levels compared with lower levels of attachment security have been found to be associated with lower levels of burden and better wellbeing [34,35,36,37,38,39]. 

### 1.3. The Current Study

Attachment towards a significant other is closely interlinked with physical proximity. In fact, spatial concepts such as closeness, distance and proximity seeking appear prominently in attachment theory, with the starting point of the theory being the presumption of a biologically based drive for physical proximity with primary caregivers [40,41,42]. In both infants and adults, physical closeness to an attachment figure is considered to provide a safe haven, fostering a fundamental sense of security and alleviating distress and anxiety during stressful situations [40,41]. In contrast, physical distance and long separation from the attachment figure are potentially disruptive and disorienting, as proximity in attachment relationships has been argued to activate a neuro-psychobiological process by which individuals reciprocally regulate one another’s mental and physical states [40,41]. Previous studies on the associations between adults’ attachment orientations and physical proximity have focused on romantic relationships [42,43,44,45]. For example, Feeney (1998) revealed that securely attached individuals have more positive perceptions of their relationship and are more likely to use viable coping strategies when dealing with physical separation [43]. Conversely, individuals with higher levels of attachment anxiety tend to experience more severe reactions of discomfort when separated from their partners and are more likely to respond to this physical separation with feelings of insecurity [43]. 

Differences in caregivers’ attachment orientations may moderate the relationship between physical proximity and caregiver burden. For example, highly anxious caregivers who live far away from the care recipient may be more stressed because of vigilance to their loved one’s accessibility. Therefore, high anxious attachment may uniquely interact with physical distance, predicting higher levels of burden for those who live further away, but not for proximate caregivers. In contrast, highly avoidant caregivers who eschew intimate relationships and seek independence may report higher levels of burden when they are physically proximate to their care recipient compared with being physically distant. Knowledge on the associations between physical proximity to the care recipient, attachment orientations and caregiver burden can provide unique and valuable insights into the translation of these aspects in the caregiving experience. Considering the interlinked nature of structural and individual factors in the context of care might help us to better understand the complexities of care provision and shed light on the experiences of caregiver burden among diverse caregiving populations. This is in line with the integrative framework proposed by Revenson et al. [6], suggesting that for studying caregiving in the illness context we should examine both the individual characteristics of caregivers and their living environment and broader context.

Therefore, in this study, we examined the relationship between physical proximity to the care recipient and caregiver burden as moderated by caregivers’ attachment orientations. Specifically, the study aimed to answer the following questions:

1. Does caregiver burden differ according to the physical proximity to the care recipient?
2. Does caregiver burden differ according to caregivers’ attachment orientations?
3. Do caregivers’ attachment orientations moderate the relationship between physical proximity and caregiver burden?

## 2. Materials and Methods

### 2.1. Study Design and Site 

An online cross-sectional survey was conducted among users of the Camoni website [46]. Launched in 2009, Camoni is the first Hebrew-language, nonprofit social support network site for patients with a chronic illness and their caregivers. Camoni—which in Hebrew means “Like me”—is comprised of 40 communities defined according to specific health conditions (e.g., heart disease, memory impairment, stroke, different types of cancer, etc.) and consists of 70,000 registered users. The website offers free-of-charge advice and medical recommendations as well as connection with other caregivers and care recipients who face the same health conditions. It includes online tools, blogs, forums, support groups and internal e-mail options. 

### 2.2. Procedure 

Ethical approval for the study was granted on 2 December 2019 by the Gertner institute review board. Data collection took part between the beginning of January 2020 and the end of March 2020. During the recruitment period, a web-based version of the self-reported questionnaire was sent to the email addresses that Camoni users had provided on registration. The survey link was also made available on the “Camoni” homepage of each of the chronic illness communities and the general caregiving community. Participation in the study was voluntary. Individuals interested to participate had to click on the survey link and provide informed written consent before taking part in the survey. Participants had the right to withdraw from the study at any time.

### 2.3. Participants

A total of 162 informal caregivers who were active users of the Camoni website gave their consent to participate in the online survey and completed the relevant demographics and outcome measures used for the current study. There were no missing data in the final sample size as participants could answer a question only if they had completed the previous ones. Participants could be primary or secondary caregivers and live with or separately from the care recipient. Eligibility criteria were as follows: (i) age 18 or over; (ii) being able to answer the survey in Hebrew; (iii) providing unpaid care to a family member or friend with a chronic illness, disability or frailty. 

### 2.4. Survey Development

The self-reported questionnaire was developed by the authors with survey items and psychometric instruments informed by the research questions and the current caregiving literature. Specifically, previous research studies investigating attachment orientations [34,36], physical proximity to the care recipient [21] and caregiver burden [34] were used as resources. A short description of the survey items used for this study is presented in the following section.

## 3. Measures

### 3.1. Sociodemographic Characteristics

A self-reported questionnaire was used to gather the sociodemographic characteristics of informal caregivers and their care recipient. These included caregivers’ age, gender, relationship status, ethnicity, religiosity, socioeconomic status, highest level of education and existing health conditions. Caregivers were also asked to specify the type of relationship with the care recipient and the care recipient’s health condition.

### 3.2. Physical Proximity to the Care Recipient 

Caregivers’ physical proximity to their care recipient was conceptualized as a continuous variable and measured by two self-reported items. First, participants were asked whether they co-reside with their care recipient: “Do you share a household with your loved one? Yes/No”. For those who replied *Yes*, proximity was coded as 0, indicating co-residence. If participants replied *No*, they were then asked to specify *in minutes* how long it takes them to arrive at the care recipient’s home: “How far (*in minutes*) is your home from the place where your loved one lives?”. For those participants, their answer was coded as the exact number of minutes reported. Travel time *in minutes* was chosen instead of miles or kilometers, because caregivers who reside a similar distance from their care recipient but use different modes of transportation may differ in the time needed to reach the care recipient’s place of residence [22]. 

### 3.3. Attachment Orientations

Caregivers’ attachment orientations were assessed using the Experiences in Close Relationships–Relationship Structures questionnaire (ECR-RS) [47]. The ECR-RS is a self-reported questionnaire designed to assess the two dimensions of attachment anxiety and avoidance using 9 items: 3 for the anxiety subscale (e.g., “I often worry that this person doesn’t really care for me”); 6 for the avoidance subscale (e.g., “I prefer not to show a partner how I feel deep down”). Caregivers were asked to rate the extent to which each item of the questionnaire was descriptive of their feelings, on a scale ranging from 1 (*not at all*) to 7 (*very much*). Scores were computed separately for each of the two subscales by averaging their item responses. Higher scores denote higher levels of attachment anxiety or avoidance. The measure has been shown to have good reliability and validity [46]. In the present study, Cronbach’s alpha was 0.74 for attachment anxiety and 0.70 for avoidance. 

### 3.4. Caregiver Burden 

Caregiver burden was measured using the Caregiver Burden Inventory (CBI) developed by Novak and Guest (1989) [48]. CBI is a 24-item multidimensional questionnaire evaluating caregiver burden with five subscales: (a) time dependence; (b) development; (c) physical burden; (d) social burden; (e) and emotional burden. Sample items include the following: (a) “I don’t get a minute of rest”; (b) “I feel that I am missing experiences in life”; (c) “I am sleep-deprived”; (d) “My functioning at work is not at the same level as it was in the past”; (e) “I am angry about our relationship”. Caregivers were asked to rate the extent to which each of the 24 items describe their feelings on a 5-point Likert scale ranging from 0 (*not at all*) to 4 (*very much*). A total burden score was obtained by summing item responses (range 0–96), with higher scores reflecting higher levels of burden. Overall, total scores nearly or slightly above 24 are considered to indicate a need of respite, whereas scores above 36 are considered to indicate a risk of burnout. The Hebrew version of the self-reported questionnaire has been previously validated using an Israeli sample of wives of war veterans, diagnosed with post-traumatic stress disorder and brain injuries [49]. Scores of the Hebrew version have previously shown high reliability [34,49]. In the present sample, Cronbach’s alpha was 0.94.

### 3.5. Statistical Analysis

Statistical analyses were conducted using IBM SPSS Statistics v25 software (IBM Corp, Armonk, NY, USA). Descriptive statistics were used for summarizing participant sociodemographic characteristics. Correlation analyses, independent-samples t-tests and one-way analysis of variance (ANOVA) were performed for examining the association of sociodemographic characteristics with CBI total score. For significant ANOVAs, Tukey post hoc analyses were performed to determine which group was significantly different from the others. Correlation analyses also examined the interrelationships of attachment orientations, physical proximity to the care recipient and caregiver burden. 

Multiple linear regression was performed to test the direct effects of attachment and physical proximity, and the interaction effects of attachment × physical proximity on caregiver burden. In step 1, selected demographic characteristics were entered as potential confounding variables. In step 2, caregiver attachment and physical proximity to the care recipient were entered. To predict the interaction effects of attachment and physical proximity on caregiver burden, two interaction terms—attachment anxiety × physical proximity; attachment avoidance × physical proximity—were entered in centered scores in step 3. All three continuous variables—attachment anxiety, avoidance and physical proximity—were mean-centered prior to creating the two interaction terms. The interaction terms were then created by taking the product of the two mean-centered main effects, respectively. Statistical significance was set at *p* < 0.05. 

## 4. Results

### 4.1. Sociodemographic Characteristics

Sociodemographic characteristics are presented in Table 1. The sample consisted of 162 Israeli Jewish caregivers, the majority of them women (67.3%), with a mean age of 57 ± 15.2 years. The caregivers’ age varied with the youngest being 18 years and the oldest 90 years. Majority of the caregivers had obtained a university degree (34.6% had a Bachelor’s degree and 24.1% had a Master’s or PhD degree) and were married or had a partner (75.9%). Consistent with the Israeli Jewish society, most caregivers described themselves as secular (70.4%) [50]. More than a third of the sample (40.8%) reported a low socioeconomic status and had a physical impairment/disability (45.1%). Caregivers were most commonly providing care for their spouse/partner (41.4%) or parent (27.2%). The largest group of the care recipients had some type of cancer or heart disease (34%), followed by care recipients with multiple chronic conditions (26.5%) and those with a physical disability or impairment (13%).

### 4.2. Univariate Analyses

Table 2 presents the means, SDs and intercorrelations between the study’s main variables. Participants displayed a relatively high mean score on the CBI as well as high variance. The mean of physical proximity between caregivers and their care recipient also varied. The minimum distance to the care recipient was 0 min, indicating co-residence ((60.4%) of the sample reported co-residence, *n* = 98), whereas the maximum was 180 min. Among caregivers who did not co-reside with the care recipient (*n* = 64), the mean of physical proximity was 30.02 ± 32.59 min. Correlational analyses showed significant positive associations between caregiver burden with attachment anxiety and avoidance but not with physical proximity. 

Independent-samples *t*-tests and ANOVAs were performed when analyzing demographic characteristics in relation to CBI total score. As illustrated in Table 3, three variables were found to be associated with caregiver burden: caregiver’s health condition; relationship to the care recipient; and care recipient’s health condition. Tukey post hoc analyses revealed that caregivers with multiple health conditions were significantly more burdened than those who reported no condition, *p* = 0.016. Furthermore, caregivers who cared for a spouse/partner (*p* = 0.013), parent *(p* < 0.001) or child (*p* = 0.004) presented with significantly higher levels of burden than those whose care recipient was a nonrelative member. Post hoc analyses did not reveal any differences on caregiver burden depending on the care recipient’s disease. Gender, education, socioeconomic and relationship status were not associated with caregiver burden. Correlation of caregiver age with burden was also not significant, *r* (160) = −0.108, *p* > 0.05. 

### 4.3. Multivariate Analyses

Table 4 shows the results of the multiple regression analysis, utilized to determine predictors of caregiver burden. In the first block of variables, the significant sociodemographic characteristics from ANOVAs, presented in Table 3, were entered as confounding variables. All three variables, caregiver’s health condition (four category variable), relationship to the care recipient (five category variable) and care recipient’s health condition (seven category variable) were recoded into three, four and six dummy variables, respectively (the number of dummy-coded variables is the number of levels minus one). Caregivers with *no health conditions or disabilities*, those who provided care to a *nonrelative member* and those who stated that their care recipient had *another condition* from the ones presented served as the reference categories for these three, four and six dummy variables, respectively. Based on former findings [51,52,53], age and gender were also factored as potential confounders in the regression model, despite not being found significant in the univariate analyses. 

The results of the regression model indicated that sociodemographic characteristics, added in step 1, accounted for a significant 22.3% of the total variance and that the model was a significant predictor of caregiver burden, *F* (15,146) = 2.99, *p* < 0.001. Specifically, caregivers with a physical impairment or disability, and those with multimorbidity, were more likely to present with higher levels of burden compared with those who had no health conditions or difficulties. Additionally, providing care to a spouse/partner, parent or child was also associated with higher burden than caring for a nonrelative member. 

In step 2 of the regression analysis, attachment anxiety, avoidance and physical proximity were factored in the model. The increase in *R*^2^ was significant and explained an additional 21.4% of variance in caregiver burden, *F* change (3143) = 17.06, *p* < 0.001. Within step 2, attachment anxiety and avoidance, were significantly and positively associated with burden. However, physical proximity to the care recipient was not. 

**Figure 1:**
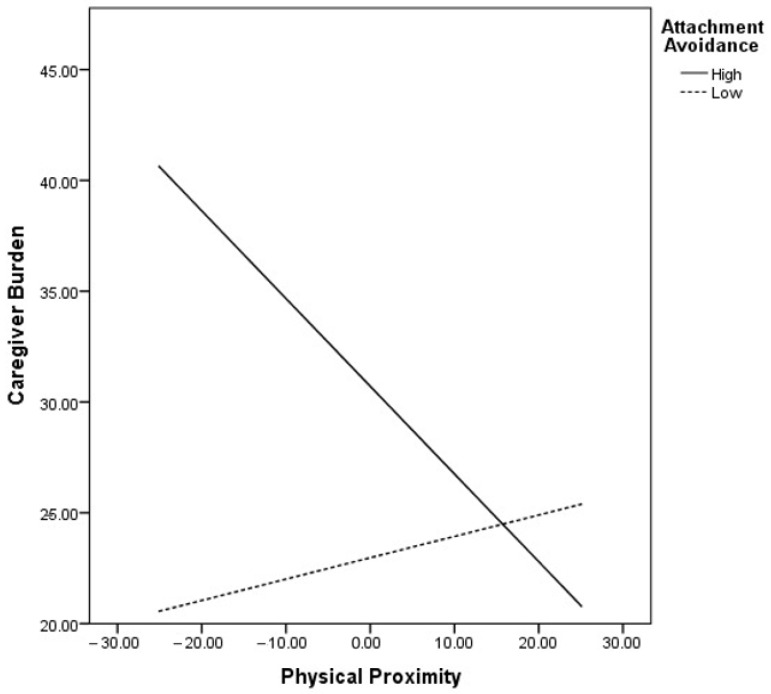
Moderating effect of attachment avoidance in the relationship between physical proximity to the care recipient and caregiver burden (mean-centered variables).

Finally, the two interaction terms, attachment anxiety × physical proximity and attachment avoidance × physical proximity, were entered into the equation. The increase in *R*^2^ was significant, explaining an additional 2.7% of variance in caregiver burden—*F* change (2141) = 3.55, *p* < 0.031. The attachment avoidance × physical proximity interaction term was significantly and negatively associated with burden whereas the anxiety × physical proximity term was not. To examine the direction of the significant interaction effect, a simple slope analysis was performed. Figure 1 presents the associations between physical proximity to the care recipient (ranging from low to high proximity) and caregiver burden, for high versus low levels of attachment avoidance (attachment avoidance was plotted at ±1 SD around the mean). Caregivers who live closer to their care recipient (including co-residing caregivers) presented higher burden at high (*B* = −0.36, *p* =0.017) but not low levels of attachment avoidance (*B* = 0.04, *p >* 0.05).

A sensitivity analysis was performed to test the moderating effect of attachment avoidance only among non-co-residing caregivers. The results were similar to those of the main analysis, revealing that caregivers who live closer to their care recipient experience greater burden at high (*B* = −0.23, *p* =0.029) but not low levels of attachment avoidance (*B* = 0.01, *p >* 0.05).

## 5. Discussion

### 5.1. Sociodemographic Characteristics

Approximately two thirds of our sample reported physical and mental health problems or multimorbidity. This finding is consistent with previous studies suggesting that caregivers are at risk of poor health [54,55,56,57,58,59]. Caregivers who had a physical impairment/disability or multiple health conditions experienced higher burden than those with no health condition. Caring for a loved one in the midst of your own deteriorating health may create additional burdens and lead to greater strain. Our results are in line with prior research indicating that caregivers’ health and burden are associated [58,59].

Participants who provided care to a spouse/partner, parent or child presented with higher levels of burden than those who cared for a nonrelative member. Family caregivers have been previously found to report greater perceived strain and lower self-rated health than nonfamily caregivers [60,61]. This might be explained by the fact that caregivers related by blood or marriage are more likely to be primary caregivers, whereas caregivers of friends and neighbors have usually less primary caretaking responsibilities [62,63,64,65]. However, in this study, the amounts of care provision and caregiver involvement were not assessed.

Prior reports have suggested that caregivers who are advanced in age [66], women caregivers [67], those with lower levels of socioeconomic status [68,69] and those caring for a care recipient with cognitive impairment [70,71], cancer [72,73,74] and heart disease [75,76] experience greater burden; however, our results did not reveal any significant differences. However, the effect of some of these sociodemographic variables may be mediated by other caregiving or care-receiving characteristics [77].

### 5.2. Main Findings

Attachment anxiety and avoidance were found to be directly and positively associated with caregiver burden, after controlling for several sociodemographic characteristics. Empirical findings have suggested that insecure attachment relates to adverse health outcomes and is predictive of emotional distress in the context of caregiving [34,35,36,37,38,39]. In line with our findings, a previous systematic review found that attachment anxiety was associated with poor mental health including increased levels of burden, depression and anxiety in 66.6% of mental health outcomes assessed across caregiving studies [36]. Anxiously attached individuals engage in more compulsive and hypervigilant forms of care provision [34,36,38]. This pattern of care is reported to be often motivated by self-focused attention and worries; it is also reported to overwhelm caregivers, physically and emotionally, as they feel deprived from having their own needs fulfilled [34,38]. The strains accompanying the caregiving role may further exacerbate those caregivers’ feelings that they are consumed by their care recipient’s needs, which must take precedence over their own [34,36].

Caregivers high on the attachment avoidance scale differed significantly from those with a lower avoidant attachment and reported higher levels of burden. The rigid self-reliance and use of cognitive and behavioral deactivating strategies in order to suppress negative affect have been previously suggested to protect avoidant caregivers from experiencing interpersonal distress and reduce the likelihood that mental health issues such as burden manifest [36]. In their review, Karantzas and colleagues [36] found that caregivers with attachment avoidance demonstrated a more controlling manner of caregiving and lacked emotional and physical closeness. Attachment avoidance was not associated with mental health outcomes in any of the reviewed studies [36]. However, as the authors highlight, the strategies that caregivers who show a high degree of attachment avoidance adopt, may not be sustainable for long periods [36]. Experiencing a significant other’s chronic illness and being placed in the role of caregiver, may make it more difficult for highly avoidant caregivers to suppress their distress and remain emotionally detached. This is in line with previous findings, revealing that in time of crisis and when facing a major stressor such a chronic health condition, deactivating strategies are not effective in regulating distressing emotions [34,78,79].

Physical proximity to the care recipient was not associated directly with burden in the correlation analyses. However, the moderation analyses revealed that the relationship between proximity and burden was moderated by attachment avoidance. Specifically, it was found that living closer to the care recipient was associated with increased levels of burden for caregivers with high but not low levels of attachment avoidance. These results support our hypothesis that comfort with interpersonal physical proximity may be related to comfort with interpersonal emotional proximity in caregiving. Consistent with the theory of attachment, caregivers may tolerate physical proximity in accordance with their attachment orientations, with those who are highly avoidant and seek emotional distance being less tolerant to physical closeness and thus more vulnerable to the pressures of caring when living closer to their care recipient [40,41,42]. Our finding is also in line with prior studies of attachment orientations and physical proximity in romantically involved adults, suggesting that more securely attached individuals are more able to seek proximity with their partner, whereas more avoidantly attached individuals may retract from their partner, both emotionally and physically [30,80,81].

Attachment anxiety did not moderate the association between physical proximity and caregiver burden. One possible explanation could be that interaction effects between attachment anxiety and physical proximity need more geographic heterogeneity and longer distances to appear. In our sample, only 7 caregivers had to travel a very long distance to reach their care recipient (>1 h), with majority of participants co-residing with the care recipient (*n* = 98) or living less than 30 min away (*n* = 47). Highly anxious DCGs who have to travel long distances may be more distressed because of vigilance to their loved one’s accessibility when compared with proximate caregivers who live closer or co-residing caregivers. Conversely, proximate and co-residing caregivers high on the attachment anxiety scale may present with comparable levels of burden as they access their care recipient in similar ways.

### 5.3. Strengths and Limitations

To the authors’ knowledge, the present study is the first to provide evidence on the moderating role of attachment orientations in the relationship between physical proximity and caregiver burden, highlighting the need for better integration of these two interlinked constructs—attachment and physical proximity—in the context of care, and serving as the foundation for future research in the field. In addition, the study includes the use of a diverse cohort of caregivers assisting care recipients with different health conditions. The sample diversity allows for broader generalization of results. Furthermore, adjustment for significant confounders, including age, gender and caregiver and care recipient characteristics, allows for more confidence in the associations reported.

Despite the strengths of this study, several limitations merit comment. First, the relatively small sample size might not be representative for exploring the role of varying degrees of physical proximities and their interaction with attachment orientations, as most caregivers co-resided with their care recipient or lived in short distances from them. Future studies should use larger samples including caregivers of various and more diverse geographic settings (e.g., countries with long travel distances) to gain a better understanding of how different proximities interact with attachment orientations and their impact on caregiver burden. Second, we conceptualized physical proximity by focusing exclusively on the travel time needed to the care recipient’s house and without assessing other—closely interlinked with time—geographic aspects such as accessibility and mode of transportation, travel costs, personal mobility problems, etc. Future research could adopt a more nuanced approach into the factors which constitute physical proximities and distances in caregiving, to adequately map these factors and enable the identification of diverse caregiving profiles. Third, our study used a cross-sectional design which precludes forming clear cause-and-effect inferences about the relationship between our study variables and caregiver burden. Future studies should use longitudinal designs to understand causal mechanisms of caregiver burden over time. Finally, the amount of care provision and caregiver involvement were not explored in this study. It is recommended that future research on physical proximities controls for the time spent in caregiving and the involvement in various care tasks, as previous studies have demonstrated significant relationships between these factors and caregiver outcomes [58,82].

## 6. Conclusions 

Despite the above limitations, the present study has important implications for clinical practice. We found that attachment anxiety and avoidance are positively associated with burden, whereas physical proximity is not. Attachment avoidance moderated the association between physical proximity and caregiver burden, with caregivers who live closer to their care recipient experiencing greater burden when high levels of avoidance are present.

Pursuant to our findings, interventions for caregivers should adopt an integrative approach, encompassing interlinked dispositional and structural factors such as caregivers’ attachment orientations and physical proximity to their care recipient. Coping strategies adopted by highly avoidant individuals who are less tolerant to physical closeness may be less effective in lowering distress and burden when facing a significant other’s health condition and being placed in the role of proximal caregiver. Support and training skills interventions considering the interlinked nature of attachment orientations with physical proximity could identify and help insecurely attached caregivers in coping with the specific care demands of proximal living arrangements—such as co-residence—to alleviate burden. In addition, highly avoidant caregivers who live with the care recipient or close to them may benefit from interventions which aim to increase attachment security and comfort with physical closeness [31,83]. Security-enhancing interventions may be also beneficial for anxiously attached caregivers, given our finding that attachment anxiety is associated with caregiver burden. Lastly, our results show that individual caregiver and care recipient characteristics—including caregivers’ health status and the type of relationship with the care recipient—should also be considered when designing support interventions as they could further inform the implementation of sustainable solutions for diverse groups of informal caregivers with different needs and burdens.

## Figures and Tables

**Table 1:** Sociodemographic characteristics.

Variable		*N*	% or *M* (*SD*)
Age		162	57.41 (15.2)
Gender	Female	109	67.3
	Male	53	32.7
Education	Secondary	29	17.9
	Post-secondary vocational education	33	20.4
	Bachelor’s degree	56	34.6
	Master/PhD Degree	39	24.1
	Other	5	3
Relationship Status	Single	17	10.5
	Married/Partner	123	75.9
	Divorced	16	9.9
	Widowed	6	3.7
Socioeconomic Status	Below average	66	40.8
	Average	48	29.6
	Above average	48	29.6
Religiosity	Secular	114	70.4
	Traditional	26	16
	Religious	22	13.6
	Other	0	0
Caregiver’s Health Condition	Physical impairment or disability	73	45.1
	Mental health problem or illness	11	6.8
	Multimorbidity	18	11.1
	No conditions or disabilities	60	37
Relationship to the Care Recipient	Spouse/Partner	67	41.4
	Parent	44	27.2
	Daughter/Son	29	17.8
	Another family member	12	7.4
	Nonrelative member	10	6.2
Care Recipient’s Health Condition	Alzheimer’s disease, dementia or any other serious memory impairment	14	8.6
	Aging	8	4.9
	Physical impairment or disability	21	13
	Cancer or heart disease	55	34
	Mental health problem or illness	7	4.4
	Multimorbidity	43	26.5
	Other	14	8.6

**Table 2:** Means, SDs and intercorrelations between caregiver burden, attachment anxiety, attachment avoidance and physical proximity to the care recipient.

Variables	*M* (*SD*)	Min	Max	*r*			
				(1)	(2)	(3)	(4)
(1) CBI	33 (21.78)	0.00	89.00				
(2) Attachment Anxiety	3.83 (1.47)	1.00	7.00	0.48 *			
(3) Attachment Avoidance	2.12 (1.53)	1.00	7.00	0.38 *	0.23 *		
(4) Physical Proximity (in Minutes)	11.86 (25.15)	0	180	0.04	0.12	−0.11	

Note. CBI—Caregiver Burden Inventory. * *p* < 0.001.

**Table 3:** Differences in caregiver burden depending on sociodemographic variables.

Variable	CBI, *M* (*SD*)	*t* or F *	*p* Value	
Gender			−1.51	0.128
	Female	34.82 (21.23)		
	Male	29.26 (22.63)		
Education			1.74	0.142
	Secondary	41.97 (23.04)		
	Post-secondary vocational education	33.64 (21.09)		
	Bachelor’s degree	31.2 (21.63)		
	Master/PhD Degree	28.95 (20.44)		
	Other	28.8 (24.6)		
Relationship Status			1.11	0.344
	Single	40.47 (21.73)		
	Married/Partner	31.33 (21.29)		
	Divorced	36.75 (25.55)		
	Widowed	36.33 (20.64)		
Socioeconomic Status			2.12	0.123
	Below average	37.05 (22.49)		
	Average	31.56 (20.67)		
	Above average	28.9 (21.36)		
Religiosity			0.58	0.560
	Secular	32.52 (21.39)		
	Traditional	31.31 (22.3)		
	Religious	37.55 (23.62)		
Caregiver’s Health Condition			3.11	0.028
	Physical impairment or disability	33.41 (22.45)		
	Mental health problem or illness	35.36 (29.7)		
	Multimorbidity	45.61(17.76)		
	No conditions or disabilities	28.3 (19.16)		
Relationship to the Care Recipient			4.76	0.001
	Spouse/Partner	31.97 (22.54)		
	Parent	39.25 (20.39)		
	Daughter/Son	36.55 (21.77)		
	Another family member	27.25 (13.25)		
	Nonrelative member	9.1 (13.03)		
Care Recipient’s Health Condition			2.53	0.023
	Alzheimer’s disease, dementia or any other serious memory impairment	43 (16.87)		
	Aging	28.88 (19.16)		
	Physical impairment or disability	31.80 (17.75)		
	Cancer or heart disease	27.64 (21.35)		
	Mental health problem or illness	49.86 (23.01)		
	Multimorbidity	37.63 (21.71)		
	Other	25.64 (26.87)		

Note. CBI—Caregiver Burden Inventory; *t* or F *—either independent-samples *t*-tests or one-way analysis of variance were performed based on the number of categories.

**Table 4:** Summary of multiple regression model for caregiver burden.

Step 1					Step 2			Step 3		
		Unstandardized Coefficients		Standardized Coefficients	Unstandardized Coefficients		Standardized Coefficients	Unstandardized Coefficients		Standardized Coefficients
		B	SE B	β	B	SE B	β	B	SE B	β
Determinant Variables										
Age		−0.15	0.12	−0.11	−0.19	0.11	−0.14	−0.17	0.1	−0.12
Gender		1.26	3.59	0.03	0.63	3.12	0.01	0.81	3.08	0.02
Caregiver’s Health Condition	Physical Impairment or Disability	7.92 *	3.74	0.18	7.42 *	3.25	0.17	7.16 *	3.2	0.16
	Mental health problem or illness	3.42	7.2	0.04	1.58	6.29	0.02	3.25	6.22	0.04
	Multimorbidity	16.39 **	5.49	0.24	15.5 **	4.93	0.22	17.37 **	4.9	0.25
Relationship to the Care Recipient	Spouse/Partner	23.06 **	7.12	0.52	17.3 **	6.33	0.39	14.46 *	6.31	0.33
	Parent	24.59 **	7.76	0.5	13.49	6.92	0.28	12.53	6.85	0.26
	Daughter/Son	24.93 **	7.78	0.44	17.99 *	6.92	0.32	14.56 *	6.92	0.26
	Another family member	12.31	9.06	0.15	8.28	7.95	0.1	5.61	7.89	0.07
Care Recipient’s Health Condition	Alzheimer’s disease, dementia or any other serious memory impairment	6.41	6.35	0.08	5.09	5.63	0.07	3.47	5.59	0.05
	Aging	−2.85	8.07	−0.03	0.06	7.06	−0.01	−1.72	6.97	−0.02
	Physical impairment or disability	−6.71	5.93	−0.1	−5.14	5.22	−0.08	−5.8	5.24	−0.09
	Cancer or heart disease	−8.01	4.53	−0.18	−6.44	3.97	−0.14	−7.13	3.94	−0.16
	Mental health problem or illness	14.3	9.48	0.13	9.94	8.48	0.09	11.5	8.61	0.11
	Multimorbidity	−6.87	7.32	−0.09	−7.1	6.35	−0.09	−6.92	6.27	−0.09
Attachment Anxiety		_	_	_	5.26 ***	1.11	0.36	5.44 ***	1.09	0.37
Attachment Avoidance		_	_	_	3.48 **	1.02	0.25	2.52 **	1.06	0.18
Physical Proximity		_	_	_	−0.03	0.06	−0.03	−0.15	0.08	−0.17
Attachment Anxiety × Physical Proximity		_	_	_	_	_	_	0.04	0.04	0.07
Attachment Avoidance × Physical Proximity		_	_	_	_	_	_	−0.16 **	0.06	−0.22
Constant		16.81	11.25		26.42	9.85		26.84	9.81	
*R* ^2^		0.223			0.437			0.464		

Note. * *p* < 0.05, ** *p* < 0.01, *** *p* < 0.001.

## Data Availability

Data will be available upon reasonable request to the corresponding author.
